# Differentiation of Human Embryonic Stem Cells into Cells with Corneal Keratocyte Phenotype

**DOI:** 10.1371/journal.pone.0056831

**Published:** 2013-02-21

**Authors:** Audrey A. Chan, Andrew J. Hertsenberg, Martha L. Funderburgh, Mary M. Mann, Yiqin Du, Katherine A. Davoli, Jocelyn Danielle Mich-Basso, Lei Yang, James L. Funderburgh

**Affiliations:** 1 Department of Ophthalmology, University of Pittsburgh School of Medicine, Pittsburgh, Pennsylvania, United States of America; 2 Department of Developmental Biology, University of Pittsburgh School of Medicine, Pittsburgh, Pennsylvania, United States of America; Wayne State University, United States of America

## Abstract

Corneal transparency depends on a unique extracellular matrix secreted by stromal keratocytes, mesenchymal cells of neural crest lineage. Derivation of keratocytes from human embryonic stem (hES) cells could elucidate the keratocyte developmental pathway and open a potential for cell-based therapy for corneal blindness. This study seeks to identify conditions inducing differentiation of pluripotent hES cells to the keratocyte lineage. Neural differentiation of hES cell line WA01(H1) was induced by co-culture with mouse PA6 fibroblasts. After 6 days of co-culture, hES cells expressing cell-surface NGFR protein (CD271, p75NTR) were isolated by immunoaffinity adsorption, and cultured as a monolayer for one week. Keratocyte phenotype was induced by substratum-independent pellet culture in serum-free medium containing ascorbate. Gene expression, examined by quantitative RT-PCR, found hES cells co-cultured with PA6 cells for 6 days to upregulate expression of neural crest genes including NGFR, SNAI1, NTRK3, SOX9, and MSX1. Isolated NGFR-expressing cells were free of PA6 feeder cells. After expansion as a monolayer, mRNAs typifying adult stromal stem cells were detected, including BMI1, KIT, NES, NOTCH1, and SIX2. When these cells were cultured as substratum-free pellets keratocyte markers AQP1, B3GNT7, PTDGS, and ALDH3A1 were upregulated. mRNA for keratocan (KERA), a cornea-specific proteoglycan, was upregulated more than 10,000 fold. Culture medium from pellets contained high molecular weight keratocan modified with keratan sulfate, a unique molecular component of corneal stroma. These results show hES cells can be induced to differentiate into keratocytes in vitro. Pluripotent stem cells, therefore, may provide a renewable source of material for development of treatment of corneal stromal opacities.

## Introduction

The cornea is an optically clear, multi-laminar tissue that functions to transmit and focus light on the retina. Connective tissue of the corneal stroma constitutes 95% of the cornea’s thickness and strength [1]. The transparency of the cornea to light depends on the unique molecular composition and organization of the extracellular matrix of the stroma, a product of keratocytes, specialized neural crest (NC) -derived mesenchymal cells. The stroma is composed of collagen fibrils stretching from limbus to limbus in parallel lamellar sheets, forming an organized, regularly spaced lattice arrangement that transmits visible light to the interior of the eye. Loss of collagen fibril organization, as occurs after trauma or infection, results in scarring and decreased transparency, sometimes leading to permanent blindness.

Currently, the only treatment for many visually-disabling corneal opacities is transplantation of corneal allografts. This therapy is highly successful, but corneal transplants are limited due to a worldwide shortage and decreasing availability of donor corneal tissue. A potential approach to address these issues is development of material suitable for stromal replacement. Currently, several models of tissue-engineered collagen-based corneal substitutes are being developed in which scaffolds are made for human keratocytes to populate [2], [3], [4]. Keratocytes, however, lose the ability to secrete and organize stromal connective tissue after expansion in vitro [5]. Therefore, there is a need for a renewable source of keratocytes, able to integrate into the scaffold and produce stromal connective tissue. Stem cells offer such a potential source for construction of biosynthetic corneal tissue [6]. Stem cells from adult tissues exhibit a limited repertoire of differentiation and typically a limited replicative lifespan in vitro, whereas stem cells derived from early embryos appear to have an unlimited lifespan and potential for differentiation to any somatic cell type. Pluripotent stem cells, therefore, offer a consistent and abundant cell source for development of bioengineering models.

Human embryonic stem (hES) cells readily differentiate into cells of neural lineage when co-cultured with the mouse fibroblast line PA6 [7]. Recently it has been shown that, during the three-week course of neural differentiation, hES cells transiently express a NC phenotype [8], [9], [10]. In the first week of co-culture the hES cells express low-affinity nerve growth factor receptor, NGFR (also known as CD271 and p75NTR) [8]. Expression of this protein is observed on migrating neural crest populations during development and is also detected on adult stem cells with NC properties [11], [12], [13]. Separation of NGFR-expressing cells before full neural differentiation isolated a population of cells with genetic, phenotypic and functional characteristics of embryonic NC cells [8].

Corneal stroma and endothelium are both tissues of NC lineage. We therefore hypothesized that differentiation of hES cells to stromal keratocytes could be effected using hES cells that have adopted a NC phenotype. In the current study we captured hES in the NC phase of their neural differentiation and induced keratocyte phenotype in pellet culture after a week-long expansion in monolayer culture. We found this sequence of culture environments to markedly upregulate expression of mRNAs characteristic of differentiated keratocytes. Furthermore the pellet-cultured cells secreted corneal-specific keratan sulfate proteoglycan.

## Materials and Methods

### hES Cell and PA6 Co-Culture

The murine stromal PA6 cell line (Riken Bioresource Center Cell Bank, Japan) was cultured on 0.1% gelatin-coated plates in 90% MEM-alpha (Life Technologies, Carlsbad, CA) containing 10% fetal bovine serum (FBS). The hES cell line WA01 (H1) was obtained from the University of Pittsburgh Stem Cell Core under license from WiCell (Madison, WI), and its use was approved by the University of Pittsburgh Human Stem Cell Research Oversight Committee. The hES cells were grown on Matrigel (BD Biosciences, Franklin Lakes, NJ) in mTeSR-1 basal medium (Stemcell Technologies, Canada) and maintained as described in previous protocols [14].

Differentiation of the hES cells into NC cells during PA6 co-culture was carried out as previously described [7] with minor modifications. Overgrown and differentiated hES colonies were identified and individually scraped off and removed from culture plates with a glass pipette. Remaining undifferentiated hES colonies were manually collected and sectioned using a StemPro EZPassage tool (Life Technologies). Remaining segmented colonies were mechanically dislodged and collected in 50 mL conical tubes, then washed and resuspended in Induction Medium (90% BHK21-medium/Glasgow modified Eagle’s medium, 2 mM glutamine, 10% knockout serum replacement, 1 mM pyruvate, 0.1 mM nonessential amino acid solution, 0.1 mM β-mercaptoethanol, 100 IU/mL penicillin, 100 µg/mL streptomycin) (all from Life Technologies) [8]. The hES colonies suspended in medium were added in a drop-wise fashion to 95% confluent PA6-cultures. The density of plating was approximately 9,000 colonies per 10 cm plate. The co-cultured plate was incubated at 37°C for 6 days without media changes.

### Immunostaining

Immunostaining was carried out on 8 µm cryostat sections of donor human corneas fixed in 3.2% paraformaldehyde overnight. Nonspecific binding was blocked with 10% heat-inactivated goat serum in phosphate buffered saline (PBS). Sections were incubated 2 hr at room temperature with 1 µg/ml primary antibodies against NGFR (Clone ME20.4, Biolegend, San Diego, CA) in 1% bovine serum albumin. After three PBS washes, anti-mouse Alexa-546 secondary antibodies and nuclear dye DAPI were added and incubated for 2 hr at ambient temperature. Samples were imaged using a confocal microscope (Olympus) with a 20× oil objective.

### Cell Isolation

Human keratocytes were isolated from central stroma of fresh human donor corneas (<48 hr from TOD) as previously described [15]. Briefly, the central cornea was excised, rinsed and incubated in 2.4 U/ml Dispase II (Roche Diagnostics, Pleasanton, CA) overnight at 4°C. Epithelial and endothelial cells were removed by dissection and debridement, and the stroma was minced into 2-mm cubes. Stroma was digested up to 3 hours at 37°C in Dulbecco’s modified Eagle’s medium (DMEM) containing 1 mg/ml collagenase type L (Sigma-Aldrich) and 0.2 mg/ml testicular hyaluronidase (Sigma-Aldrich). Cells were harvested by centrifugation and immediately lysed for RNA as described below.

### Quantitative Reverse Transcription-polymerase Chain Reaction (qPCR)

hES cell samples were collected at days 2, 4, 6, and 8 of PA6 co-culture and lysed in 0.35 ml RLT buffer for RNA isolation using the RNeasy mini kit (Qiagen, Valencia, CA). The RNA was treated with DNase I and concentrated by ethanol precipitation. First strand cDNA was prepared from 400 ng RNA by reverse transcription using Super Script First Strand Synthesis System for RT-PCR (Life Technologies) as described [16]. Quantitative RT-PCR (qPCR) was performed using SYBR Green reagents (Fisher Scientific Inc.) with primers as shown in Table 1 or with previously reported primers for adult human corneal stem cells and keratocytes [15], [17]. Sequences of primers for the human NC genes were compared to their mouse homologues in the NCBI mouse RefSeq mRNA library with BLAST (http://blast.ncbi.nlm.nih.gov/) to rule out amplification of mouse cDNA by these primers. qPCR with these primers on cDNA from PA6 cells confirmed their non-reactivity with murine RNA. Amplification was 40 cycles of 15 sec at 95°C and 1 min at 60°C after initial incubation at 95°C for 10 min. Total reaction volume was 20 µL, including Maxima SYBR Green qPCR Master Mix (containing Maxima Hot Start Tac DNA polymerase, ROX, MgCl_2_, and nucleotides, Fermentas, Fisher Scientific) with cDNA transcribed from 20 ng of RNA and 0.2 µM forward and reverse primers. The StepOne Real-Time PCR System (Applied Biosystems) was used to generate a dissociation curve for each reaction. Mean threshold cycle number (Ct) of triplicate reactions was determined by StepOne Software v2.2.2 and compared to the mean Ct value for 18S for the same cDNA and expressed as a power of 2 to calculate relative cDNA abundance.

**Table 1:** PCR Primer Sequences.

Gene (GenBank mRNA)	Forward Primer	Reverse Primer
GAPDH (NM_002046)	TGTTGCCATCAATGACCCCTT	CTCCACGACGTACTCAGCG
NGFR (NM_002507)	CCTACGGCTACTACCAGGATG	CACACGGTGTTCTGCTTGTC
NTRK3 (NM_001007156)	TCCGTCAGGGACACAACTG	GCACACTCCATAGAACTTGACA
SOX9 (NM_000346)	GCCAGGTGCTCAAAGGCTA	TCTCGTTCAGAAGTCTCCAGAG
SNAI1 (NM_005985)	AATCGGAAGCCTAACTACAGCG	GTCCCAGATGAGCATTGGCA
SLUG (NM_003068)	AAGCATTTCAACGCCTCCAAA	AGGATCTCTGGTTGTGGTATGAC
MSX1 (NM_002448)	CTCCGCAAACACAAGACGAAC	CACATGGGCCGTGTAGAGTC
Mouse Tbp1 (NM_013684)	AGAACAACAGCCTTCCACCTTATG	CAAGTTTACAGCCAAGATTCACGG

### Isolation of NGFR+ Cells

On day 7 of PA6 co-culture, the hES colonies were dislodged with Accutase (Life Technologies) and triturated by pipetting into a single-cell suspension. After rinsing twice, the cells were filtered through a 70 µm cell strainer to remove any clumps and counted. The cells were washed twice in PBS containing 0.5% bovine serum albumin and ethylene-diamine-tetraacetic acid, 0.1 mM, then resuspended in 0.1 mL of the same buffer. After adding Fc blocking reagent (Miltenyi Biotec, Auburn, CA), the cells were fluorescently labeled by incubating with APC-labeled anti-NGFR antibody, washed, and incubated with magnetic beads covalently linked to anti-APC antibodies (Miltenyi Biotec). The MACS Cell Separation system (Miltenyi Biotec) was then used to separate NGFR+ magnetically labeled cells according to the manufacturer’s instructions. **Flow-through** and **Bound** cell populations were collected from the MACS columns and analyzed as to NGFR by flow cytometry and for NC gene expression by qPCR. For flow cytometry, 10^5^ cells in 0.1 ml PBS were incubated with 2 µg APC-labeled antibody to NGFR (Miltenyi Biotec) or with APC-labeled nonspecific isotype control antibody along with Fixable Violet Live/Dead stain (Life Technologies) for 30 min on ice. Cells were rinsed by centrifugation and then fixed in 2% paraformaldehyde in PBS. Staining was determined by flow cytometry on a BD Biosciences FACS Aria III flow cytometer.

### Cell Expansion and Pellet Culture

NGFR+(Bound) cells were cultured as monolayers in 10 cm tissue culture dishes coated with FNC (AthenaES, Baltimore, MD) in MEM-alpha with 10% FBS or alternately on plates coated with poly-L-ornithine/laminin/fibronectin [9] in N2 Medium [DME/F12 medium (Sigma) with N2 supplement (Life Technologies), 10 ng/ml FGF2 (Sigma) and 10 ng/ml EGF (Sigma)]. Because of the presence of abundant PA6 cells, flow-through cells were not further cultured. After 7 days, the cultured Bound cells were collected for qPCR analysis and also transferred to pellet culture to induce keratocyte-differentiation [17]. Briefly, 1.8×10^5^ cells were collected in 15-mL conical tubes and centrifuged at 1500 rpm for 5 minutes to form pellets. The pellets were cultured 2% FBS in DME-F12 medium and after 2 days transferred to keratocyte differentiation medium (KDM): Advanced DMEM with 10 ng/mL FGF2, and 0.1 mM ascorbic acid-2-phosphate [17]. Although cells in pellets maintained viability based on staining with Calcein AM, the pellets were difficult to disperse and were not passaged.

For protein expression analysis, NGFR+ cells were cultured for two weeks as pellets in DMEM/F-12, with 1 mM ascorbate-2-phosphate, ITS (Gibco), 0.1 mM non-essential amino acids, 10 ng/mL FGF2, and antibiotics as above. Proteoglycans were isolated from conditioned media by ion exchange chromatography as described previously [18], dialyzed, dried, and biotin labeled using sulfosuccinimidyl-6-(biotinamido) hexanoate (Sulfo-NHS-LC-Biotin, Fisher Scientific) at 2 mg/ml in 0.1 M NaHCO_3_, 1 hr at room temperature. Cornea-specific keratan sulfate proteoglycans were immune precipitated with a polyclonal antibody to keratocan [5], [18] or a monoclonal antibody to keratan sulfate, J19 [19], bound to protein G-magnetic beads (Dynabeads, Life Technologies). Keratan sulfate on half of each sample was digested with endo-beta-galactosidase [5] (QA-Bio, Palm Desert, CA) 0.05 U/ml, 2 hr at 37°C. Digested and undigested samples were separated by electrophoresis on 4%–20% SDS-PAGE gel and transferred to a PVDF membrane. Membranes were probed with streptavidin-IR700 dye and imaged on a LiCor Odyssey Imaging System [20].

## Results

### Selecting Keratocyte Precursor Cells by NGFR Expression

**Figure 1:**
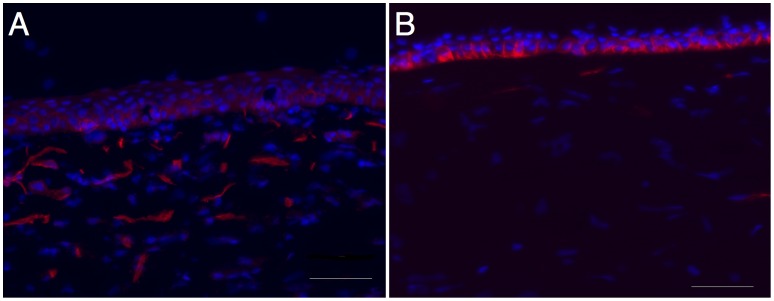
Limbal stroma cells express NGFR protein. Cryosections of cornea from a 34 yr old donor were immunostained with antibody to NGFR (p75ntr) protein(red) and counterstained with DAPI (blue) to show nuclei. (A) Shows anterior stroma and epithelium at the limbus. (B) Shows central cornea. Bars in the images indicate 50 µm.

We and others have shown that the limbal (peripheral) region of the corneal stroma contains mesenchymal cells with stem cell properties [6]. These cells have the ability to differentiate to keratocytes in vitro and in vivo [17], [21], [22]. Consistent with the neural crest origin of the stroma, limbal stromal cells express several proteins characteristic of neural precursor and neural crest cells including nestin and Six2 [17]. The cell surface low affinity nerve growth receptor (NGFR) is expressed on migrating embryonic neural crest cells as well as a number of adult stem cells, particularly those with neural crest character [12], [13], [23]. NGFR has also been detected in limbal epithelial and stromal cells of human cornea [24], [25]. In the immunostaining in Fig. 1 we confirmed the observation that cells in the limbal stroma express NGFR (Fig. 1A) and found that few or no cells in the central stroma stained for NGFR (Fig. 1B). Because hES cells exhibit transient expression of NGFR when induced to differentiate to the neural lineage [8], we adopted the rationale that isolation of hES cells expressing NGFR might provide a source of cells with the potential to differentiate to keratocytes.

### Isolation and Characterization of hES-derived NC Cells

**Figure 2:**
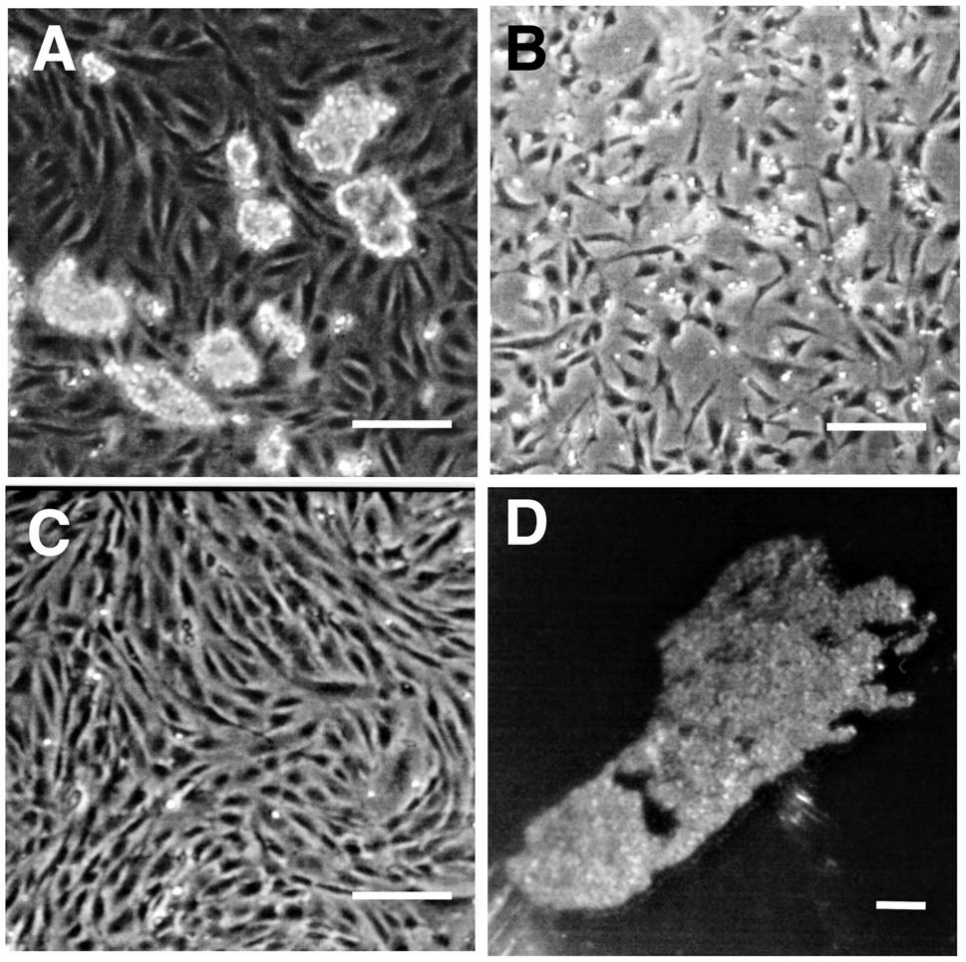
Culture of hES cells. ( A) Shows colonies of hES cells cultured on monolayers of PA6 mouse cells to induce neural differentiation. (B) NGFR+ derived hES cells after 6 days of co-culture were cultured as monolayers in serum-free N2 medium as described in Methods. The cells remain small and exhibit polygonal morphology. (C) NGFR+ derived hES cells are cultured as a monolayer in serum-containing alpha-MEM medium as described in Methods. The cells form aligned, spindle-shaped confluent monolayers. (D) Pellet from alpha-MEM cultured cells after 2-week culture. Cells are small, tightly packed and difficult to disperse. Bars show 200 µm.

**Figure 3:**
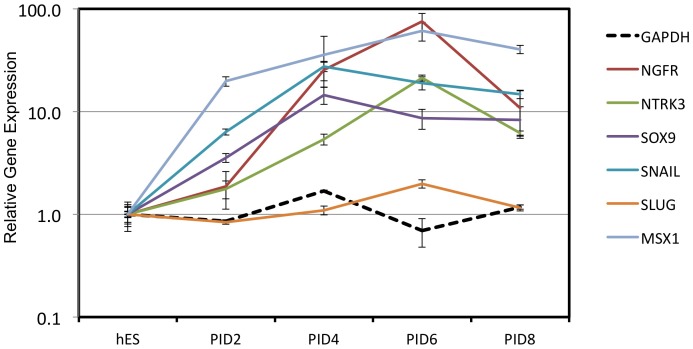
Co-culture with PA6 cells induces upregulation of neural crest gene expression in hES cells. hES cells were co-cultured with PA6 as described in Methods. RNA was isolated at post-induction days (PID) 2, 4, 6, and 8. Expression of characteristic neural crest (NC) marker genes was determined by qPCR as described in Methods using human-specific primers (Table 2). Expression levels are calculated relative to untreated hES cells (hES = 1). Error bars show the standard deviation (S.D.) of triplicate analyses.

When hES cells are co-cultured with mouse embryonic fibroblast PA6 cells (Fig. 2A) they differentiate to neural cells [26]. Early in this process the hES cells transiently express a NC phenotype [8]. We found that expression of several characteristic NC genes (NGFR, SNAI1, NTRK3, SOX9, and MSX1) was upregulated after two days of the co-culture (Fig. 3). Consistent with previous reports, expression of these NC genes plateaued at days 6–8, after which the cells transitioned into neural cell phenotypes [2], [4], [15].

**Figure 4:**
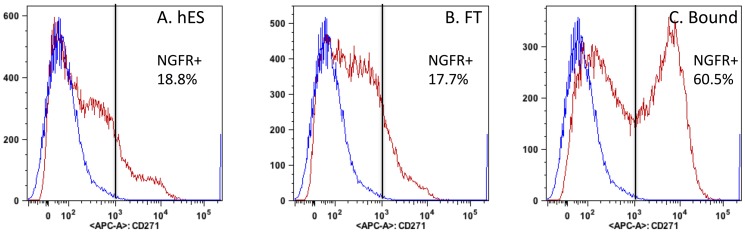
Magnetic-activated cell sorting (MACS) enriches for a population of NGFR+ cells. Single cell suspensions of hES cells after 6 days of co-culture with PA6 cells were stained with APC-labeled NGFR antibody and analyzed by flow cytometry either before or after separation of NGFR-positive cells by MACS columns as described in Methods. The blue trace shows the cells stained with non-specific isotype-matched control antibody. (**A**) Unfractionated hES+PA6 co-culture. (**B**) Flow-through, cells not bound to the NGFR MACS column. (**C**) NGFR+ cells bound and released from MACS column. The vertical bar marks the population containing <0.1% non-specific control cells (blue trace). The calculated percentages of the population (red trace) with positive staining are listed on the graph.

From 6-day induced hES cells, a population of cells expressing the cell surface NC protein NGFR was selected using magnetic beads attached to anti-NGFR antibody (Bound Cells). Cells expressing lower levels of NGFR (Flow Through) were also collected. Flow cytometry of these populations (Fig. 4) showed that the affinity procedure enriched for cells with the NGFR cell surface marker, increasing its abundance 4-fold in the Bound-cell population compared to starting material. 4.9×10^6^ cells were recovered from a starting population of 7.8×10^7^ total (hES+PA6) cells representing about 6% of the total cells. Viability of the Bound cells was >95%.

**Figure 5:**
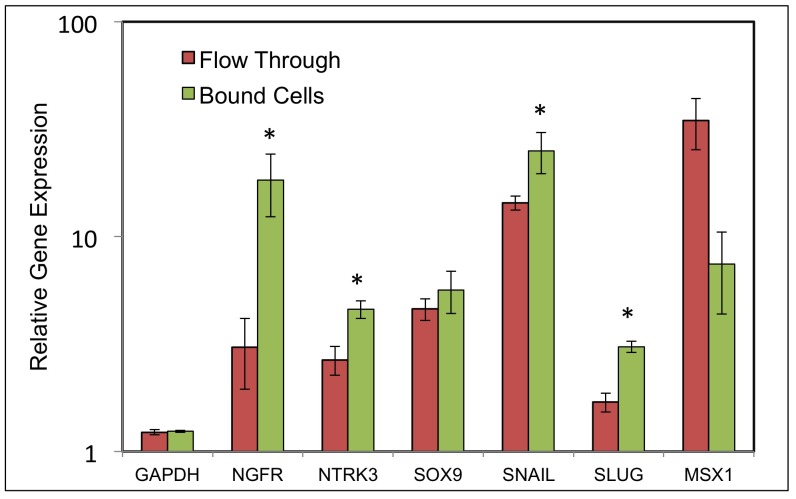
Expression of neural crest gene markers in co-cultured hES cells after magnetic separation. Expression of human NGFR, NTRK3, SOX9, SNAI1, SLUG and MSX1 was determined in 6-day PA6-cocultured hES cells after separation of NGFR+ (Bound) and NGFR- (Flow through) by MACS columns as described under Methods. Expression levels were calculated by qPCR relative to hES cells as described in Fig 3. (hES expression = 1). Error bars show S.D. of triplicate analyses. Asterisks show significantly (p<0.05) increased expression in bound cells as determined by Student’s t-test.

Quantification of NC gene expression in the isolated (Bound) cell population (Fig. 5) found NGFR expression to be enriched 6-fold compared to Flow-though cells**.** Expression of other NC marker genes (NTRK3, SNAI1, and SLUG) was significantly enriched in the NGFR+ cells, but two NC markers (SOX9 and MSX1) did not appear to be specifically associated with the NGFR+ cell population.

Because the cells introduced to the MACS columns represented a mixture of hES and murine PA6 feeder layer cells, it was important to verify that PA6 cells were not collected in the NGFR+ fraction. Presence of mRNA for a constitutively expressed mouse gene TBP1 [27] was compared using qPCR from the starting cells and the cells after isolation. The expression relative to the pure PA6 line is shown in Table 2. By this measure, the recovered NGFR+ cells were found to be >99.99% human, similar to the pure hES cell cultures. This proportion remained after expansion and passage of these cells. Therefore, magnetic-activated cell sorting is an effective way of isolating a NGFR+ population of cells from PA6 co-cultured treated hES cell culture without contamination from the murine feeder layer.

**Table 2:** Expression of mouse mRNA in isolated NGFR+ Cells.

Cells	Mouse Tbp1
PA6 (mouse cells)	100
hES (human cells)	0.002
NGFR+ MEM-FBS p0	0.001
NGFR+ MEM-FBS p1	0.0005
NGRF+ N2 p0	0.002

### Keratocyte Differentiation of hES-derived NGFR+ Cell Population

**Figure 6:**
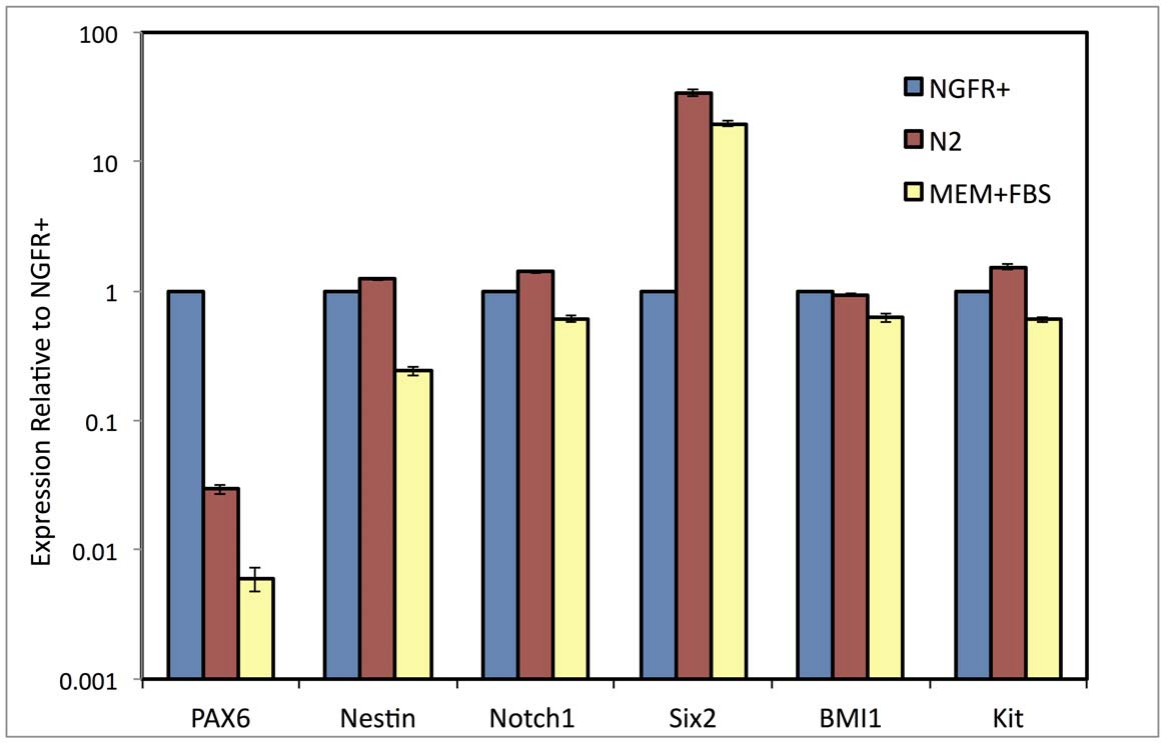
Expression of adult stem cell markers during monolayer culture. Expression of genes distinguishing adult corneal stem cells from keratocytes: PAX6, NES, NOTCH1, SIX2, BMI1, and KIT, was examined in the NGFR+ cells and in these cells after culture of the cells one week as monolayers in MEM-FBS or in N2 media as described in Methods. Gene expression was determined by qPCR relative to NGFR+ cells (set = 1). Error bars show S.D. of triplicate analyses.

Adult stem cells isolated from the human corneal stroma exhibit a phenotype different from differentiated keratocytes, but with similarities to mesenchymal stem cells from bone marrow [15], [16], [17]. These cells can be distinguished from keratocytes by expression of several stem cell-associated genes, and will differentiate into keratocytes when cultured as substratum-free floating pellets in serum-free medium containing ascorbic acid-2-phosphate and FGF2 [17]. Our initial attempts to maintain the isolated NGFR+ cells directly in pellet cultures led to cell death, but we found that expansion of these cells in monolayer culture (as shown in Fig. 2B and 2C) maintained their viability. In Fig. 6, we compared isolated NGFR+ cells and the subsequent monolayer cultures for expression of six genes previously identified as abundant in human corneal stromal stem cells, (Pax6, Nestin, Kit, Notch1, Six2, BMI1) [17]. All six genes were expressed in the NGFR+ cells and in culture, expression was maintained for all but PAX6 which was decreased by >90% in the cultured cells. Expansion in serum-free conditions in the presence of EGF, FGF2 and N2 supplement (N2 medium, Fig. 2B) was marginally better at maintaining stem cell gene expression than culture in 10% fetal bovine serum in MEM-alpha medium (MEM-FBS, Fig. 2C). Passage in MEM-FBS reduced the adult stem cell expression even further (not shown).

**Figure 7:**
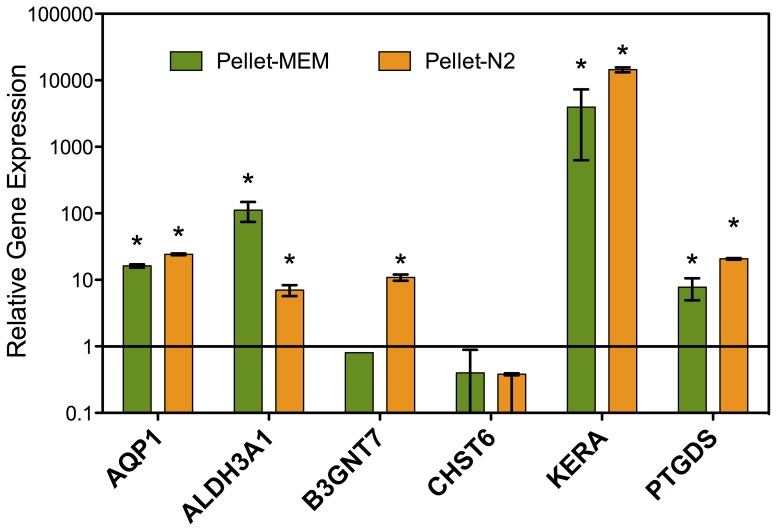
Upregulation of keratocyte-specific gene expression in pellet cultures. Expression of six genes, previously identified as up-regulated during keratocyte differentiation, was determined after 2 weeks in pellet cultures derived from either MEM+FBS or N2 monolayer cultures as described in Methods. Gene expression is calculated relative to the NGFR+ derived hES cells. Error bars represent S.D. of triplicates. All genes were significantly (p<0.05) upregulated in pellets compared to NGFR+ cells except for CHST6. Asterisks show cases in which pellet culture induced a significant (p<0.05) increase in gene expression compared to the monolayers cultures.

Cells from the N2 and MEM-FBS monolayer cultures were transferred to differentiation medium as pellets (Fig. 2D) for two weeks and assayed for expression of six genes that are highly expressed in keratocytes. As shown in Fig. 7, aquaporin-1 (AQP1) was increased 24-fold, PTGDS 20-fold, B3GNT7 10-fold, and ALDH3A1 100-fold when compared with the NGFR+ cells. Most notably, expression of keratocan (KERA), a cornea-specific proteoglycan present in stromal extracellular matrix, was increased over 10,000-fold.

**Figure 8:**
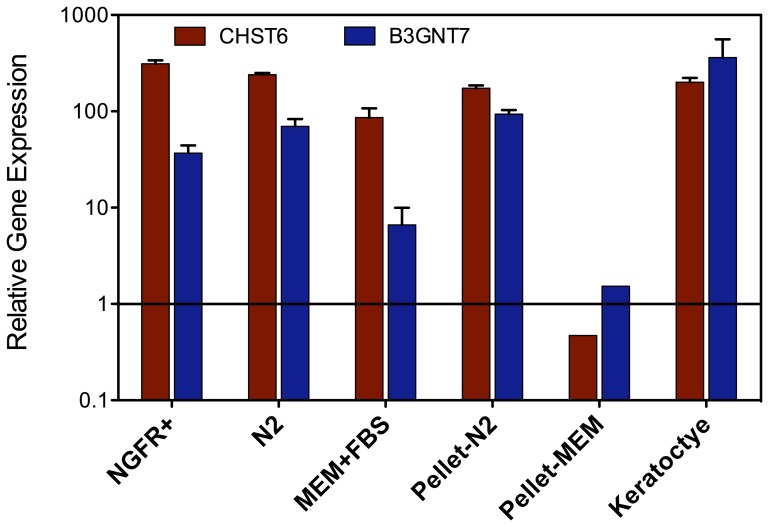
Expression levels of CHST6 and B3GNT7. Expression of CHST6 and B3GNT7 in the hES-derived cells was compared relative to that of human corneal fibroblasts (cells that do not secrete keratan sulfate) and to freshly isolated stromal keratocytes as described in Methods. Levels of both genes are comparable in NGFR+ cells to those in keratocytes but are reduced in the presence of FBS. Cell cultures are those described in Methods. Error bars show S.D. of triplicate analyses.

The most characteristic molecular identifiers of keratocytes are the keratan sulfate proteoglycans [28]. These are a group of three proteins, one of which being keratocan, modified by highly sulfated keratan sulfate glycosaminoglycan chains. Biosynthesis of corneal keratan sulfate is reported to require two cornea-specific enzymes, beta3-GnT7 (EC 2.4.1), a glycosyltransferase, and corneal N-acetylglucosamine 6-sulfotransferase (EC 2.8.2.17) [29]. Messenger RNA for keratocan (KERA) and for these two enzymes (B3GNT7, CHST6) are all markedly increased when adult stem cells differentiate to keratocytes [22], so it seemed curious that the CHST6 showed little upregulation as hES-derived cells began expressing keratocan. To better understand this phenomenon we compared expression of the biosynthetic genes in hES-derived cells with that of human corneal fibroblasts (HCF), cells that do not synthesize keratan sulfate, and with freshly isolated uncultured human keratocytes [15]. In Fig. 8 we found that the NGFR+ cells as well as the monolayer cultures all express high levels of the keratan sulfate sulfotransferase (CHST6) mRNA, almost equivalent to that in uncultured keratocytes and that the glycosyltransferase (B3GNT7) mRNA was also highly abundant compared to the level in corneal fibroblasts. Cells in MEM-FBS had reduced mRNA abundance for these genes, and when cultured as pellets, expressed little more than the HCF. Pellet cultures from cells expanded in N2 medium, however, showed levels of expression almost identical to those in keratocytes. These results suggest that CHST6 and B3GNT7 may not be upregulated in pellet culture because they are already expressed at high levels by the NGFR+ cells.

**Figure 9:**
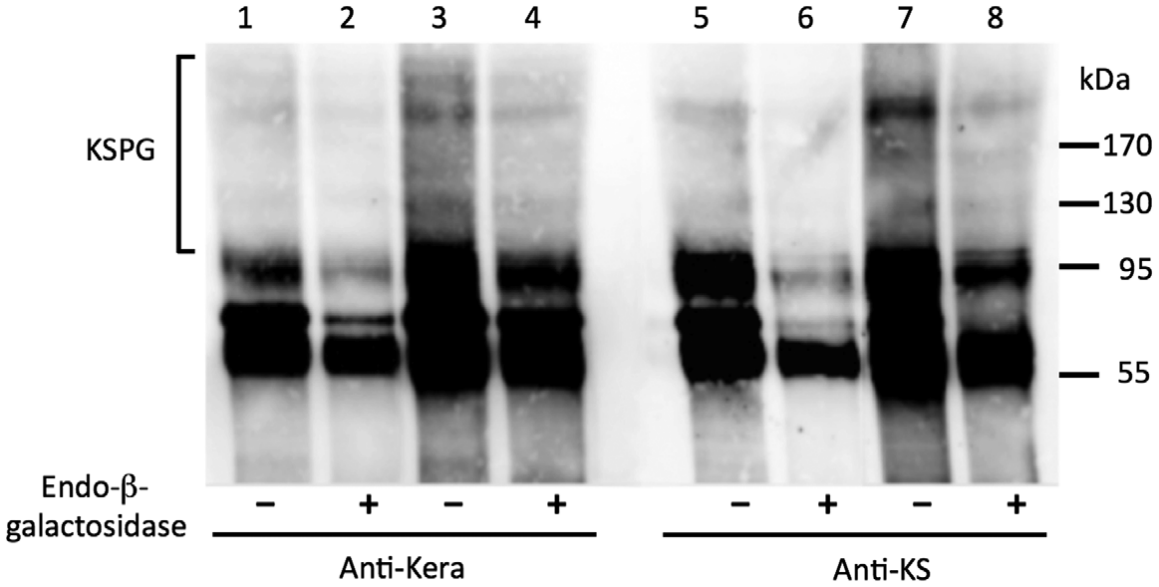
Secretion of corneal keratan sulfate proteoglycans by pellet cultures. Proteoglycans were isolated from culture medium before (Lanes 1,2,5,6) or after (3,4,7,8) three-week incubation with hES pellets. The proteoglycan fractions were biotin- labeled and immune-precipitated with antibodies against keratocan (anti-Kera) (lanes 1–4) or keratan sulfate glycosaminoglycan (anti-KS) (lanes 5–8). Half of each sample was digested with endo-ß-galactosidase (as described under Methods) to hydrolyze keratan sulfate, and samples were separated by SDS-PAGE, transferred to PVDF membranes and biotinylated proteins detected with avidin-labeled infrared dye as described in Methods. Presence of biotinylated proteins migrating as a broad, heterogeneous >100 band typical of keratan sulfate proteoglycan (KSPG - bracket on the left) was present in lanes 3 and 7. Sensitivity of this material to digestion with keratan sulfate-specific glycosidase (lanes 4 and 8) demonstrates presence of keratocan-linked keratan sulfate, a unique keratocyte biosynthetic product.

Secretion of high molecular weight keratan sulfate proteoglycans (KSPG) is a unique phenotypic property of keratocytes and is required for corneal transparency. High expression of KERA, B3GNT7 and CHST6 suggests that these specialized proteoglycans may be produced by the hES-derived pellet cultures. Antibodies to keratocan precipitated a high molecular weight (>100 kDa) heterogeneous protein from pellet-conditioned culture media (Fig. 9 lane 3). This material was sensitive to digestion by endo-beta-galactosidase (Fig. 9, lane 4), a keratan sulfate-degrading enzyme. Presence of the keratan sulfate in this fraction was confirmed by immune precipitation with anti-keratan sulfate monoclonal antibody J19 and subsequent digestion with endo-beta-galactosidase (Fig. 9 Lanes 7 & 8). These results demonstrate secretion in the pellet cultures of molecular components of corneal stroma that represent unique biosynthetic products of differentiated keratocytes.

## Discussion

In this study, we show that hES-derived cells expressing NC marker genes can be induced to differentiate into cells expressing keratocyte-specific genes and extracellular matrix components. Using a well-established co-culture procedure (Fig. 2A) that induces neuronal differentiation in H1-hES cells, we selected a population of NGFR-expressing cells. As previously reported, these cells express several characteristic NC marker genes. The isolated NGFR+ cells were expanded in monolayer cultures during which time they continued expression of several genes we previously identified in adult corneal stromal stem cells [17]. The monolayer cells were then incubated as free-floating pellets, a condition we previously found to induce the keratocyte phenotype in adult stem cells [17]. In the pellets, expression of genes that characterize the phenotype of keratocytes was detected, including KERA, AQP1, ALDH3A1, CHST6, B3GNT7 and PTGDS. To confirm the phenotype we demonstrated the presence of keratan sulfate proteoglycans containing both keratocan and high-molecular weight keratan sulfate in conditioned media from the pellet cultures. These matrix components are tissue-specific biosynthetic products of keratocytes present only in corneal stroma. Previously, keratocan production has been induced in various multipotent stem cells including adult adipose-derived stem cells [19], corneal stromal stem cells [17], and bone marrow mesenchymal stem cells [30]. The current results provide convincing evidence that stromal extracellular matrix can also be produced by pluripotent stem cells.

An important aspect of the selection process was the use of NGFR-expressing cells. Selection of NGFR+ cells allowed isolation of a population with neural crest phenotype, free of mouse feeder cells. NGFR is a cell surface protein expressed mostly in migrating NC cells in embryos [11]. Stromal keratocytes are derived from NC, and adult human cornea was recently reported to express mRNA for NGFR [31]. Our data in Fig. 1 confirm previous reports [24], [25] that NGFR protein is present on cells in the limbal stromal cells. Furthermore, this figure adds new information that little NGFR protein is present in the central corneal stroma. Our observation that hES-derived cells expressing NGFR have the potential to become keratocytes is consistent with the idea that the NGFR+ cells in limbal stroma may be keratocyte progenitor cells.

Immobilized anti-NGFR antibodies have been previously used to select NC–like populations from hES cells during neural differentiation [8]. This approach clearly enriched for a NGFR+ population, but according to Fig. 4 the isolated population contained a substantial sub-population of NGFR- cells as well. In the future we may obtain a more potent population by multiple rounds of selection or by fluorescence-activated cell sorting (FACS). NC gene expression by these cells suggests the differentiating hES cells were heterogeneous because not all of the NC gene expression segregated equally with the NGFR+ cells. NGFR mRNA was enriched by at least 6-fold in the NGFR+ selected cells but other NC markers much less so. One advantage of the selection process was that it completely eliminated mouse PA6 feeder cells (Table 2).

We found the NGFR+ population unable to differentiate to keratocytes directly and introduced an intermediate monolayer culture of the NGFR+ cells. During this culture, expression of genes previously found to be expressed in stromal stem cells, particularly that of PAX6, was reduced. Fetal bovine serum and expansion of the cells beyond a single passage appeared to be detrimental to their ultimate ability to differentiate. Although this culture stage appears to be essential, it seems likely that additional optimization of conditions of this stage of the process might ultimately provide a more efficient differentiation.

The ability of adult cells to differentiate to keratocytes appears to require the presence of soluble ascorbate and culture in an environment allowing formation of a three-dimensional cell construct [17], [19], [22], [32], [33], [34], [35]. We originally found that in serum-free medium containing ascorbate-2-phosphate, insulin, and FGF2, primary keratocytes form free-floating spheres that release from the plastic surface, adapting a highly differentiated phenotype [33]. Spherical aggregates of adult stem cells from cornea, adipose, and trabecular meshwork tissues formed by centrifuging the cells into a pellet also express keratocyte genes and organize a cornea-like extracellular matrix [17], [19], [32]. Based on these studies, we believe that expression of an array of keratocyte-specific genes by hES-derived NGFR+ cells cultured under these same conditions provides a strong argument that these cells have become functional keratocytes.

This argument was bolstered by the detection (in Fig. 9) of corneal keratan sulfate proteoglycans secreted by the pellets. These data document secretion of a high molecular weight (>100 kDa) heterogeneous protein that precipitates with antibodies to keratocan and keratan sulfate and is also degraded by a glycosidase that breaks down keratan sulfate. Since this molecular form of proteoglycan is uniquely secreted by keratocytes in vivo, we believe this experiment provides incontrovertible evidence that the hES-derived cells have adopted a function previously only observed in corneal keratocytes.

Production of keratocytes from pluripotent cells has significant implications for cell-based therapy and tissue engineering for treatment of corneal diseases. Based on these results, pluripotent hES cells could represent a consistent, inexhaustible source of tissue for the surgical treatment of severe corneal opacities. Furthermore, induced pluripotent stem cells derived from adult somatic cells could be used in place of human embryonic stem cells to provide autologous material for bioengineered corneal matrix or for direct cell-based therapy having a decreased risk of rejection and in greater supply than donor tissue.

In summary, this study has developed methodology to induce differentiation of hES cells into cells with a gene-expression phenotype similar to that of adult human keratocytes. This method may prove useful in the ongoing development of cell-based treatment for corneal blindness.

## References

[1] Morishige N, Takagi Y, Chikama T, Takahara A, Nishida T (2011) Three-dimensional analysis of collagen lamellae in the anterior stroma of the human cornea visualized by second harmonic generation imaging microscopy. Invest Ophthalmol Vis Sci 52: 911–915. 20881300 10.1167/iovs.10-5657

[2] Vrana NE, Builles N, Justin V, Bednarz J, Pellegrini G, et al (2008) Development of a reconstructed cornea from collagen-chondroitin sulfate foams and human cell cultures. Invest Ophthalmol Vis Sci 49: 5325–5331. 18708614 10.1167/iovs.07-1599

[3] Torbet J, Malbouyres M, Builles N, Justin V, Roulet M, et al (2007) Tissue engineering of the cornea: orthogonal scaffold of magnetically aligned collagen lamellae for corneal stroma reconstruction. Conf Proc IEEE Eng Med Biol Soc 2007: 6400. 18003486 10.1109/IEMBS.2007.4353820

[4] Doillon CJ, Watsky MA, Hakim M, Wang J, Munger R, et al (2003) A collagen-based scaffold for a tissue engineered human cornea: physical and physiological properties. Int J Artif Organs 26: 764–773. 14521175 10.1177/039139880302600810

[5] Long CJ, Roth MR, Tasheva ES, Funderburgh M, Smit R, et al (2000) Fibroblast growth factor-2 promotes keratan sulfate proteoglycan expression by keratocytes in vitro. J Biol Chem 275: 13918–13923. 10788517 10.1074/jbc.275.18.13918

[6] Pinnamaneni N, Funderburgh JL (2012) Stem Cells in the Corneal Stroma. Stem Cells. 10.1002/stem.1100 PMC3580383 22489057

[7] Pomp O, Brokhman I, Ben-Dor I, Reubinoff B, Goldstein RS (2005) Generation of peripheral sensory and sympathetic neurons and neural crest cells from human embryonic stem cells. Stem Cells 23: 923–930. 15883233 10.1634/stemcells.2005-0038

[8] Jiang X, Gwye Y, McKeown SJ, Bronner-Fraser M, Lutzko C, et al (2009) Isolation and characterization of neural crest stem cells derived from in vitro-differentiated human embryonic stem cells. Stem Cells Dev 18: 1059–1070. 19099373 10.1089/scd.2008.0362 PMC4606969

[9] Lee G, Chambers SM, Tomishima MJ, Studer L (2010) Derivation of neural crest cells from human pluripotent stem cells. Nat Protoc 5: 688–701. 20360764 10.1038/nprot.2010.35

[10] Lee G, Kim H, Elkabetz Y, Al Shamy G, Panagiotakos G, et al (2007) Isolation and directed differentiation of neural crest stem cells derived from human embryonic stem cells. Nat Biotechnol 25: 1468–1475. 18037878 10.1038/nbt1365

[11] Betters E, Liu Y, Kjaeldgaard A, Sundstrom E, Garcia-Castro MI (2010) Analysis of early human neural crest development. Dev Biol 344: 578–592. 20478300 10.1016/j.ydbio.2010.05.012 PMC2927129

[12] Abe S, Hamada K, Miura M, Yamaguchi S (2012) Neural crest stem cell property of apical pulp cells derived from human developing tooth. Cell Biol Int 36: 927–936. 22731688 10.1042/CBI20110506

[13] Vaculik C, Schuster C, Bauer W, Iram N, Pfisterer K, et al (2012) Human dermis harbors distinct mesenchymal stromal cell subsets. J Invest Dermatol 132: 563–574. 22048731 10.1038/jid.2011.355 PMC3278768

[14] Ludwig TE, Bergendahl V, Levenstein ME, Yu J, Probasco MD, et al (2006) Feeder-independent culture of human embryonic stem cells. Nat Methods 3: 637–646. 16862139 10.1038/nmeth902

[15] Du Y, Funderburgh ML, Mann MM, SundarRaj N, Funderburgh JL (2005) Multipotent stem cells in human corneal stroma. Stem Cells 23: 1266–1275. 16051989 10.1634/stemcells.2004-0256 PMC1941788

[16] Funderburgh ML, Du Y, Mann MM, SundarRaj N, Funderburgh JL (2005) PAX6 expression identifies progenitor cells for corneal keratocytes. FASEB J 19: 1371–1373. 15901670 10.1096/fj.04-2770fje PMC2876310

[17] Du Y, Sundarraj N, Funderburgh ML, Harvey SA, Birk DE, et al (2007) Secretion and organization of a cornea-like tissue in vitro by stem cells from human corneal stroma. Invest Ophthalmol Vis Sci 48: 5038–5045. 17962455 10.1167/iovs.07-0587 PMC2874676

[18] Funderburgh JL, Mann MM, Funderburgh ML (2003) Keratocyte phenotype mediates proteoglycan structure: a role for fibroblasts in corneal fibrosis. J Biol Chem 278: 45629–45637. 12933807 10.1074/jbc.M303292200 PMC2877919

[19] Du Y, Roh DS, Funderburgh ML, Mann MM, Marra KG, et al (2010) Adipose-derived stem cells differentiate to keratocytes in vitro. Mol Vis 16: 2680–2689. 21179234 PMC3002955

[20] Roh DS, Funderburgh JL (2011) Rapid changes in connexin-43 in response to genotoxic stress stabilize cell-cell communication in corneal endothelium. Invest Ophthalmol Vis Sci 52: 5174–5182. 21666237 10.1167/iovs.11-7272 PMC3176070

[21] Du Y, Carlson EC, Funderburgh ML, Birk DE, Pearlman E, et al (2009) Stem cell therapy restores transparency to defective murine corneas. Stem Cells 27: 1635–1642. 19544455 10.1002/stem.91 PMC2877374

[22] Wu J, Du Y, Watkins SC, Funderburgh JL, Wagner WR (2012) The engineering of organized human corneal tissue through the spatial guidance of corneal stromal stem cells. Biomaterials 33: 1343–1352. 22078813 10.1016/j.biomaterials.2011.10.055 PMC3254093

[23] Cox G, Boxall SA, Giannoudis PV, Buckley CT, Roshdy T, et al (2012) High abundance of CD271(+) multipotential stromal cells (MSCs) in intramedullary cavities of long bones. Bone 50: 510–517. 21807134 10.1016/j.bone.2011.07.016 PMC3268250

[24] Qi H, Li DQ, Shine HD, Chen Z, Yoon KC, et al (2008) Nerve growth factor and its receptor TrkA serve as potential markers for human corneal epithelial progenitor cells. Exp Eye Res 86: 34–40. 17980361 10.1016/j.exer.2007.09.003 PMC2198932

[25] Qi H, Chuang EY, Yoon KC, de Paiva CS, Shine HD, et al (2007) Patterned expression of neurotrophic factors and receptors in human limbal and corneal regions. Mol Vis 13: 1934–1941. 17982417 PMC2185513

[26] Turksen K (2006) Human Embryonic Stem Cell Protocols: Humana Press. 10.1007/978-1-4939-2668-8 26478938

[27] Ren S, Zhang F, Li C, Jia C, Li S, et al (2010) Selection of housekeeping genes for use in quantitative reverse transcription PCR. Mol Vis 16: 1076–1086. 20596249 PMC2893048

[28] Funderburgh JL (2000) Keratan sulfate: structure, biosynthesis, and function. Glycobiology 10: 951–958. 11030741 10.1093/glycob/10.10.951

[29] Kitayama K, Hayashida Y, Nishida K, Akama TO (2007) Enzymes responsible for synthesis of corneal keratan sulfate glycosaminoglycans. J Biol Chem 282: 30085–30096. 17690104 10.1074/jbc.M703695200

[30] Liu H, Zhang J, Liu CY, Hayashi Y, Kao WW (2011) Bone marrow mesenchymal stem cells can differentiate and assume corneal keratocyte phenotype. J Cell Mol Med. 10.1111/j.1582-4934.2011.01418.x PMC4365890 21883890

[31] Lambiase A, Merlo D, Mollinari C, Bonini P, Rinaldi AM, et al (2005) Molecular basis for keratoconus: lack of TrkA expression and its transcriptional repression by Sp3. Proc Natl Acad Sci U S A 102: 16795–16800. 16275928 10.1073/pnas.0508516102 PMC1283852

[32] Du Y, Roh DS, Mann MM, Funderburgh ML, Funderburgh JL, et al (2012) Multipotent Stem Cells from Trabecular Meshwork Become Phagocytic TM Cells. Invest Ophthalmol Vis Sci 53: 1566–1575. 22297497 10.1167/iovs.11-9134 PMC3339918

[33] Funderburgh ML, Mann MM, Funderburgh JL (2008) Keratocyte phenotype is enhanced in the absence of attachment to the substratum. Mol Vis 14: 308–317. 18334944 PMC2255023

[34] Guo X, Hutcheon AE, Melotti SA, Zieske JD, Trinkaus-Randall V, et al (2007) Morphologic characterization of organized extracellular matrix deposition by ascorbic acid-stimulated human corneal fibroblasts. Invest Ophthalmol Vis Sci 48: 4050–4060. 17724187 10.1167/iovs.06-1216 PMC4961093

[35] Karamichos D, Guo XQ, Hutcheon AE, Zieske JD (2010) Human corneal fibrosis: an in vitro model. Invest Ophthalmol Vis Sci 51: 1382–1388. 19875671 10.1167/iovs.09-3860 PMC2868432

